# Rituximab in Mucous Membrane Pemphigoid: Current Evidence and Clinical Perspectives

**DOI:** 10.3390/healthcare14183062

**Published:** 2026-09-17

**Authors:** Liliana Gabriela Popa, Calin Giurcaneanu, Valeria Coviltir, Cristina Popescu, Selda Ali, Marina Cozma, Olguta Anca Orzan

**Affiliations:** 1 Department of Dermatology, Carol Davila University of Medicine and Pharmacy, No. 37 Dionisie Lupu Street, 030167 Bucharest, Romania; 2 Department of Dermatology, Elias Emergency University Hospital, No. 17 Marasti Bd, 011461 Bucharest, Romania; 3 Department of Ophthalmology, Carol Davila University of Medicine and Pharmacy, No. 37 Dionisie Lupu Street, 030167 Bucharest, Romania; 4 Department of Ophthalmology, Clinical Institute of Ophthalmological Emergencies Prof. Dr. Mircea Olteanu, No. 1, Alexandru Lahovari Square, 010464 Bucharest, Romania; 5 Department of Allergology and Clinical Immunology, Carol Davila University of Medicine and Pharmacy, No. 37 Dionisie Lupu Street, 030167 Bucharest, Romania; 6 Department of Allergology, Dr. Carol Davila Nephrology Clinical Hospital, No. 4 Grivitei Bd., 010731 Bucharest, Romania

**Keywords:** mucous membrane pemphigoid, autoimmune blistering diseases, rituximab, B-cell depletion

## Abstract

**Background:** Mucous membrane pemphigoid (MMP) is a chronic, debilitating disease associated with significant morbidity. Progressive fibrosis of affected mucosal surfaces may result in irreversible complications, including blindness secondary to cicatricial conjunctivitis, esophageal strictures, laryngeal stenosis causing airway compromise, and genitourinary scarring with functional impairment. The management of MMP is still extremely challenging. **Objectives:** To assess the demographic and clinical characteristics of patients with MMP diagnosed and treated in our clinic during the past 5 years, and to analyze the evolution of the disease under conventional and biologic treatments. **Methods:** We conducted a retrospective study of patients with MMP treated in our clinic between January 2021 and May 2026. **Results:** We identified eight patients with MMP. The time from onset of the disease until diagnosis ranged from 1 month to 10 years. Seven patients had already developed significant complications of the disease, including esophageal stenosis, vision impairment, or unilateral vision loss. The disease exhibited progressive worsening in all patients, despite treatment with glucocorticoids, immunosuppressants, or immunomodulatory medication. Rituximab was administered to six patients. Although disease control was achieved in all cases, continuation of systemic conventional treatment was required. The mean time to significant clinical improvement was 2 months. One patient suffered a severe relapse 12 months following rituximab administration and required additional doses. **Conclusions:** Based on our experience, we support the early use of rituximab in high-risk patients with MMP. Prompt diagnosis and early, aggressive treatment in the inflammatory phase are essential to prevent scarring.

## 1. Introduction

The earliest clinical description consistent with mucous membrane pemphigoid (MMP) dates back to 1794, when Wichmann reported a bullous disorder affecting both the skin and mucous membranes, resulting in bilateral blindness and complete denudation of the oral cavity and lips [1]. Over the following decades, only a limited number of similar cases were documented. Ocular manifestations, in particular, were described under alternative designations, including “syndesmitis degenerativa”, proposed by Stellwag von Carion in 1870 [2], and “essential shrinking of the conjunctiva”, introduced by von Kries in 1878 [3]. Although these authors recognized that this condition differed from pemphigus, it continued to be regarded as one of its clinical forms for several decades.

A major conceptual advance took place in 1911, when Thost distinguished the “acute” mucosal involvement seen in pemphigus vulgaris and pemphigus foliaceus from a separate chronic disorder characterized by progressive scarring of the affected mucosal surfaces and a benign, non-fatal clinical course [4]. In recognition of these distinctive features, he introduced the term “benign mucous membrane pemphigus” in 1917 [5]. Subsequently, the terms “ocular pemphigus” and “pemphigus conjunctivae” came into general use for cases limited to the conjunctiva, whereas “benign mucous membrane pemphigus” was reserved for patients with involvement of additional mucosal sites, with or without concomitant skin disease [6].

The distinction between MMP and pemphigus was ultimately established by Lever, whose histopathologic and cytologic studies of 153 patients with bullous disorders demonstrated the absence of acantholysis and the subepidermal blister formation in MMP [7]. Based on these findings, he continued to use the term “benign mucous membrane pemphigoid” and recognized it as a distinct clinicopathologic entity.

Although MMP is only rarely life-threatening, it is a chronic, debilitating disease associated with significant morbidity. Progressive fibrosis of affected mucosal surfaces may result in irreversible complications, including blindness secondary to cicatrizing conjunctivitis, esophageal strictures with dysphagia, laryngeal stenosis causing airway compromise, and genitourinary scarring associated with functional impairment. As clinical experience accumulated, it became evident that the term “benign” underestimated the disease’s potentially devastating sequelae. Therefore, during the 1980s, the disease was generally referred to as “cicatricial pemphigoid”.

In 2002, the First International Consensus on Mucous Membrane Pemphigoid [8] recommended the term “mucous membrane pemphigoid” for the heterogeneous group of autoimmune subepithelial blistering disorders that primarily involve mucosal surfaces, with or without evident scarring, caused by the production of immunoglobulin (Ig)G or IgA autoantibodies that target a series of antigens of the basal membrane zone (BMZ). Essential diagnostic criteria were established and included a typical clinical picture, i.e., the presence of vesicles, bullae, and erosions on any mucous membrane with or without scarring and skin involvement, and positive direct immunofluorescence studies showing continuous deposits of IgG, IgA, and/or complement C3 at the BMZ. Due to the fragility of the mucosal epithelium, the histopathologic examination may fail to demonstrate the characteristic subepithelial blister accompanied by a variable inflammatory infiltrate within the superficial lamina propria. Indirect immunofluorescence studies can provide valuable diagnostic support, but their utility is limited by the diversity of the target autoantigens, which may localize to either the epidermal or dermal side of the BMZ, and the fact that circulating autoantibodies are undetectable in a substantial proportion of patients with MMP [9,10,11]. Consequently, neither the presence of characteristic light microscopic findings nor positive indirect immunofluorescence results has been considered an absolute diagnostic criterion [8].

MMP is a rare disease, with reported annual incidence rates ranging from 0.7 to 2 cases per million [9,12,13,14,15]. Onset usually takes place in the seventh to ninth decade of life. Pediatric MMP is exceptionally rare [16,17]. MMP shows a predilection for the female gender, with the estimated female/male ratio being 1.5–2 [18]. No differences in incidence have been observed among racial groups [18].

**Figure 1:**
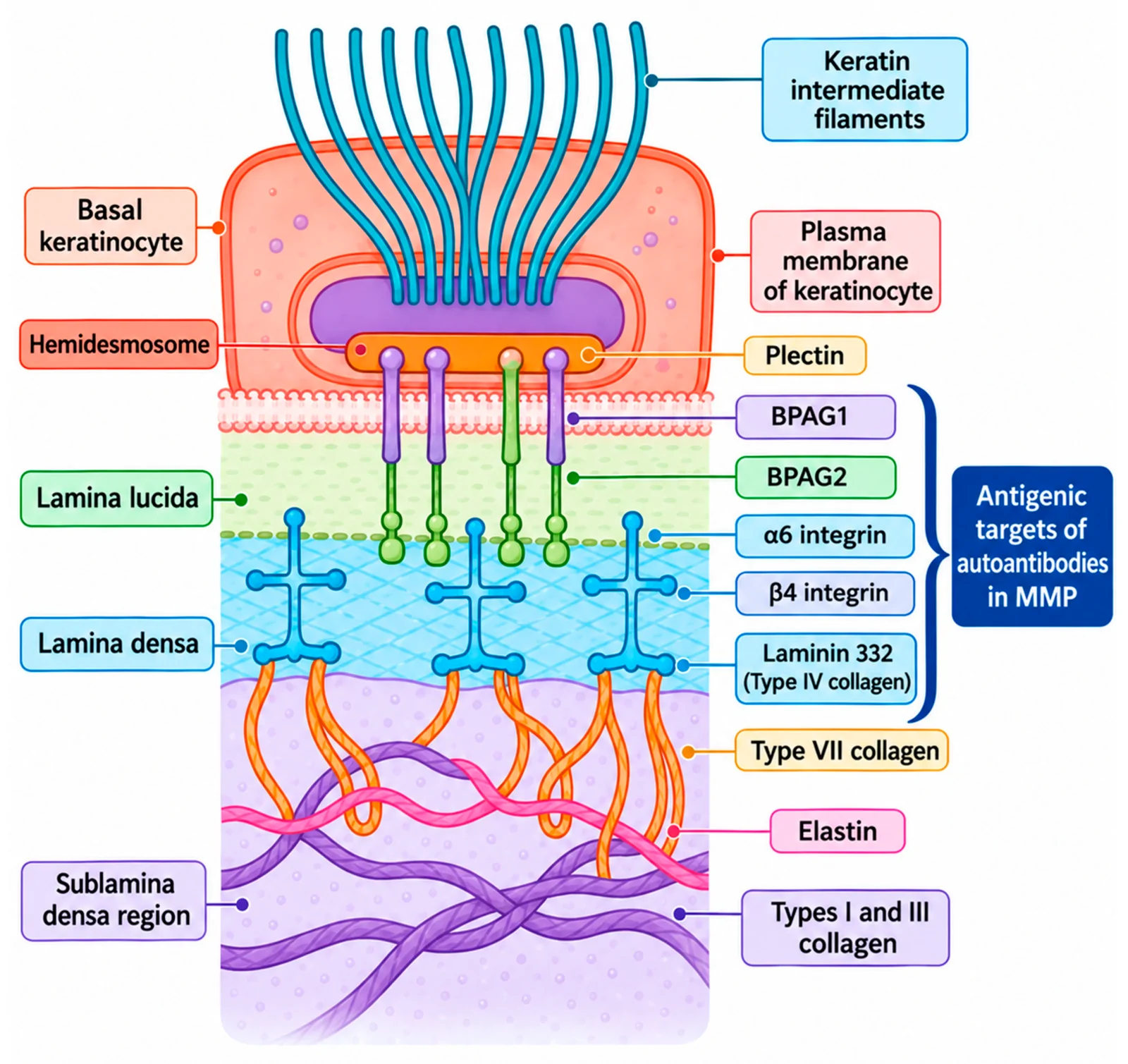
The structure of the basal membrane zone, illustrating the main autoantigens targeted in MMP.

Certain haplotypes, including HLA-DQB1*0301, DRB*04, and DRB*11, have been linked to the development of MMP [19,20,21]. However, the cause of the loss of tolerance to structural components of the BMZ remains elusive. Although autoantibodies against bullous pemphigoid antigen (BP) 180, BP 230, α6β4 integrin, laminin 332, laminin 6, and type VII collagen have been detected in patients with MMP, to date, a pathogenic role has only been demonstrated for anti-laminin 332 and anti-α6β4 integrin antibodies (Figure 1) [9,22,23,24,25]. An underlying malignancy was detected in up to 30% of patients with MMP with circulating anti-laminin 332 antibodies [9,26,27,28,29,30]. This is explained by the presence of laminin 332 in the extracellular matrix of various neoplasms and its important role in tumor cell adhesion and motility [31]. A plausible hypothesis is that immune cells within the tumor-associated inflammatory infiltrate initiate a humoral immune response against laminin 332, with cross-reactivity to laminin 332 expressed at the BMZ [32,33]. No correlation between other autoantibodies and MMP clinical subtypes has been proven. These observations suggest the involvement of additional pathogenic mechanisms in the development of the disease. Following the binding of autoantibodies to self-antigens, innate and adaptive immune cells infiltrate the site. The inflammatory infiltrate is composed of neutrophils, eosinophils, macrophages, and CD4+T cells, which release reactive oxygen species, proteolytic enzymes, cytokines—predominantly interleukin (IL)-4, IL-13, and tumor necrosis factor (TNF)-α—and growth factors, mainly transforming growth factor (TGF)-β [9,11,34,35,36]. The latter leads to the activation of fibroblasts and, implicitly, to fibrosis.

The majority of patients develop painful oropharyngeal ulcerations, aphthous lesions, or desquamative gingivitis that render oral hygiene, alimentation, and hydration difficult. The laryngeal and esophageal mucosae are affected in 12% and 5% of patients with MMP, respectively [37]. Chronic inflammation of the laryngeal mucosa triggers dysphonia and stridor, while esophageal lesions generate dysphagia. Progressive tissue damage and scarring in these sites may have potentially fatal consequences, including laryngeal stenosis with impaired airway patency and esophageal stenosis with nutritional compromise, risk of aspiration pneumonia or esophageal rupture, and subsequent mediastinitis [38]. Ophthalmic involvement is frequent, occurring in up to 70% of patients with MMP. It usually has a unilateral onset and initially manifests as conjunctivitis. With advancing disease, the conjunctival fornices shorten and fibrous bands develop, culminating in symblepharon and ankyloblepharon. Trichiasis due to cicatricial entropion, together with severe ocular dryness caused by limbal stem cell insufficiency, results in corneal ulcerations, neovascularization, gradual keratinization, and opacification, ultimately leading to corneal perforation and vision loss [39,40]. Nasopharyngeal lesions may also occur, resulting in nasal discharge, epistaxis, and crusting. Genitourinary and anorectal involvement may lead to strictures, causing dyspareunia, dysuria, fecal incontinence, or rectal bleeding [9,19,26]. Approximately 30% of patients also present with skin lesions.

The clinical picture and disease severity are highly variable. In 1999, during an expert consensus meeting, MMP was classified into a “low risk” clinical form, in which the lesions are confined to the oral mucosa, and a “high risk” variant, displaying involvement of other mucosae, a marked propensity for scar formation, and an unfavorable prognosis even with aggressive treatment [8]. Either form may feature skin lesions, including erythematous plaques and bullae, which leave ulcerations that often heal with atrophic scarring [19,41].

MMP should be suspected in any patient with recurrent mucosal blisters and erosions. Other autoimmune bullous disorders, as well as lichen planus, lupus erythematosus, erythema multiforme minor or major, and infectious diseases, should be considered in the differential diagnosis.

The management of MMP is still extremely challenging and requires a multidisciplinary approach. To our knowledge, no randomized controlled trial has been published to date, and only a limited body of clinical trial evidence is available regarding the efficacy of various therapeutic agents due to the rarity of the disease. The location, extent, and severity of the lesions determine the choice of treatment. Proper, gentle hygiene of the affected tissue and avoidance of trauma are mandatory. Local anti-inflammatory agents, including corticosteroids, topical calcineurin inhibitors, and anesthetics, are indicated in all patients. Topical ophthalmic cyclosporine, serum-based tear substitutes, and lubricants are also frequently recommended in ocular MMP [10].

In patients with low-risk MMP, immunomodulatory drugs such as dapsone 25–200 mg daily or doxycycline 200 mg daily—which may be associated with nicotinamide—are the first-line treatment. If disease control is not achieved, systemic glucocorticoids in low-to-medium doses, combined with immunosuppressive agents—like azathioprine 1–2.5 mg/kg daily, methotrexate 7–15 mg weekly, or mycophenolate mofetil 2–2.5 g daily—are the mainstay of therapy [10,41,42,43]. Rituximab, an anti-CD20 monoclonal antibody, is considered a third-line treatment in refractory low-risk MMP according to the 2021 European Academy of Dermatology and Venereology (EADV) Guidelines [10].

Aggressive systemic therapy with high-dose glucocorticoids, combined with corticosteroid-sparing immunosuppressants—such as azathioprine, mycophenolate mofetil, or cyclophosphamide 2 mg/kg daily administered orally or 0.5–1 g monthly intravenous pulse therapy—should be promptly initiated in high-risk forms of MMP in order to halt disease progression and prevent irreversible complications [8,41,42,43]. The addition of dapsone to this treatment regimen is advised [42]. Plasmapheresis/immunoadsorption, high-dose intravenous immunoglobulin (IVIG), and B-cell depletion with rituximab are employed in recalcitrant severe MMP [20,42,43,44]. Anti-TNFα agents represent fourth-line treatments [10,45]. However, response to treatment is unpredictable, and currently available therapies fail to induce disease control in a substantial proportion of patients [45].

We wish to present our clinical experience with rituximab in the management of MMP, discuss considerations for its optimal use, and outline the most appropriate management strategies.

## 2. Materials and Methods

We conducted a retrospective observational study on patients with MMP treated in our clinic between January 2021 and May 2026. The main objectives of this study were to assess the demographic and clinical characteristics of patients with MMP diagnosed and treated in our tertiary-level clinic during the past 5 years and to analyze the evolution of the disease under conventional and biologic treatments.

## 3. Results

We identified eight patients diagnosed with MMP based on clinical, histopathologic, and direct and indirect immunofluorescence examinations. Following diagnosis, a multidisciplinary team was established to oversee patient care, including a dermatologist, an ophthalmologist, a dentist, an otolaryngologist, and a gastroenterologist, depending on the specific features of each case. In patients with ocular involvement, we used the staging system proposed by Foster et al. [46], detailed in Table 1.

All patients were Caucasians. Overall, 7 of the 8 patients were female. The mean age was 66 ± 13 years. The time from the onset of the disease until diagnosis ranged from 1 month to 10 years, with the mean period being 33 months. The disease was limited to mucosal sites in five patients, whereas mucocutaneous involvement was observed in three patients. Oropharyngeal involvement was present in five patients and ocular involvement in six. All patients displayed severe ophthalmic lesions, which represented stage IV according to Foster’s staging system. Laryngeal, esophageal, and anogenital involvement was detected in one patient each. More than three mucosal sites were affected in two patients. At presentation in our clinic, seven patients had already developed significant complications of the disease, including esophageal stenosis that required multiple dilations, vision impairment, and unilateral vision loss due to structural compromise, necessitating enucleation. The disease was active, progressively worsening in all patients, despite combined systemic treatment with glucocorticoids, immunosuppressants or immunomodulatory medication, and topical anti-inflammatory treatment. Circulating antibodies directed against the BP180 antigen were detected in seven patients. The patient characteristics are presented in Table 2.

Rituximab was administered in six patients according to the pemphigus vulgaris protocol: two intravenous infusions of 1000 mg, administered two weeks apart. The decision to initiate rituximab was based on disease severity, progression under standard treatment, and threat to critical functions. Although all eight patients were eligible, only six patients ultimately received rituximab due to financial constraints, as rituximab is not approved for MMP in our country and therefore not reimbursed. Before initiation of rituximab treatment, all patients underwent screening for infections, including viral serology (hepatitis B surface antigen, antibodies against hepatitis B core antigen, hepatitis C virus, and human immunodeficiency virus), and tuberculosis screening (interferon-gamma release assay and chest X-ray), which yielded negative results except in the patient with known chronic hepatitis B infection. Baseline immunoglobulin levels were within normal limits in all patients. Premedication with methylprednisolone 100 mg intravenous infusion, oral acetaminophen, and an oral nonsedating antihistamine was administered 30 min prior to each infusion. The patients were closely monitored throughout rituximab administration and during the following 24 h. Rituximab treatment was generally well tolerated. Two patients experienced mild infusion-related reactions, including transitory pruritus and headache. One patient developed a hypersensitivity reaction to rituximab, manifesting as acute urticaria and dry cough, but successful desensitization was later carried out, and the patient was able to complete the initiation regimen. All patients continued treatment with corticosteroids and immunosuppressants, such as azathioprine and methotrexate, or immunomodulatory agents, mainly dapsone. Local treatment was also continued and consisted of corticosteroids and anesthetics in an oral adhesive base for the oral cavity and antiseptics and corticosteroids for the skin lesions. Management of ocular manifestations included the use of artificial tears and ocular lubricants, topical corticosteroids, cyclosporine, therapeutic contact lenses, lacrimal duct plugs, and repeated eyelash epilation for trichiasis.

The patients presented for monthly follow-up visits. Clinical improvement was defined as the absence of new blisters or erosions/ulcerations, together with a decrease in clinical inflammation. Relapse was defined as the reappearance of new blisters or erosions/ulcerations, and/or an increase in clinical inflammation. Biological monitoring included a complete blood count and metabolic panel, inflammatory markers, and immunoglobulin levels. The outcomes were assessed retrospectively based on the information documented in the patients’ medical records. This included data from the patients’ clinical files, as well as the findings and assessments provided through multidisciplinary evaluation. These documented clinical assessments were reviewed to determine the relevant outcomes for each patient.

All patients treated with rituximab showed clinical improvement. Although disease control was achieved, continuation of the systemic conventional treatment was required in all cases. The mean time to clinical improvement was 2 months. After a quiescent period of 12 months, patient no. 6 developed a severe relapse and required an additional course of rituximab. Rituximab was not administered according to a predefined fixed retreatment schedule. Although retreatment at 6-month intervals was considered, the main factor limiting its use was the financial burden of the treatment, as patients were unable to afford regular rituximab administration at 6-month intervals. Consequently, decisions regarding subsequent treatment were made on an individual basis, according to the patient’s clinical course, treatment response, and ability to undergo further treatment. The outcomes following rituximab treatment are detailed in Table 3.

The two patients who did not receive rituximab had a fluctuating disease course, characterized by periods of exacerbation that required adjustment of the doses of conventional therapies and/or the addition of other conventional treatment modalities.

The main limitations of our study are the small number of patients and the lack of longitudinal data on relevant biological parameters, such as CD20+ B-cell counts and autoantibody titers, due to its retrospective nature. Nevertheless, the rarity of this condition and the difficulties associated with its diagnosis have limited the availability of large case series, with few substantial cohorts reported to date. Therefore, our series adds to the sparse body of evidence on the efficacy of biologic treatment in this uncommon disease.

The absence of a control group is another important limitation, as the effects of rituximab cannot be fully isolated from those of concomitant treatments and other potential confounding factors.

The lack of a standardized response assessment and the possibility of referral bias represent additional limitations of our study. These limitations are, to some extent, inherent to the retrospective study design, in which data are dependent on the information available in patients’ medical records and on the clinical assessment performed at the time of evaluation.

## 4. Discussion

**Figure 2:**
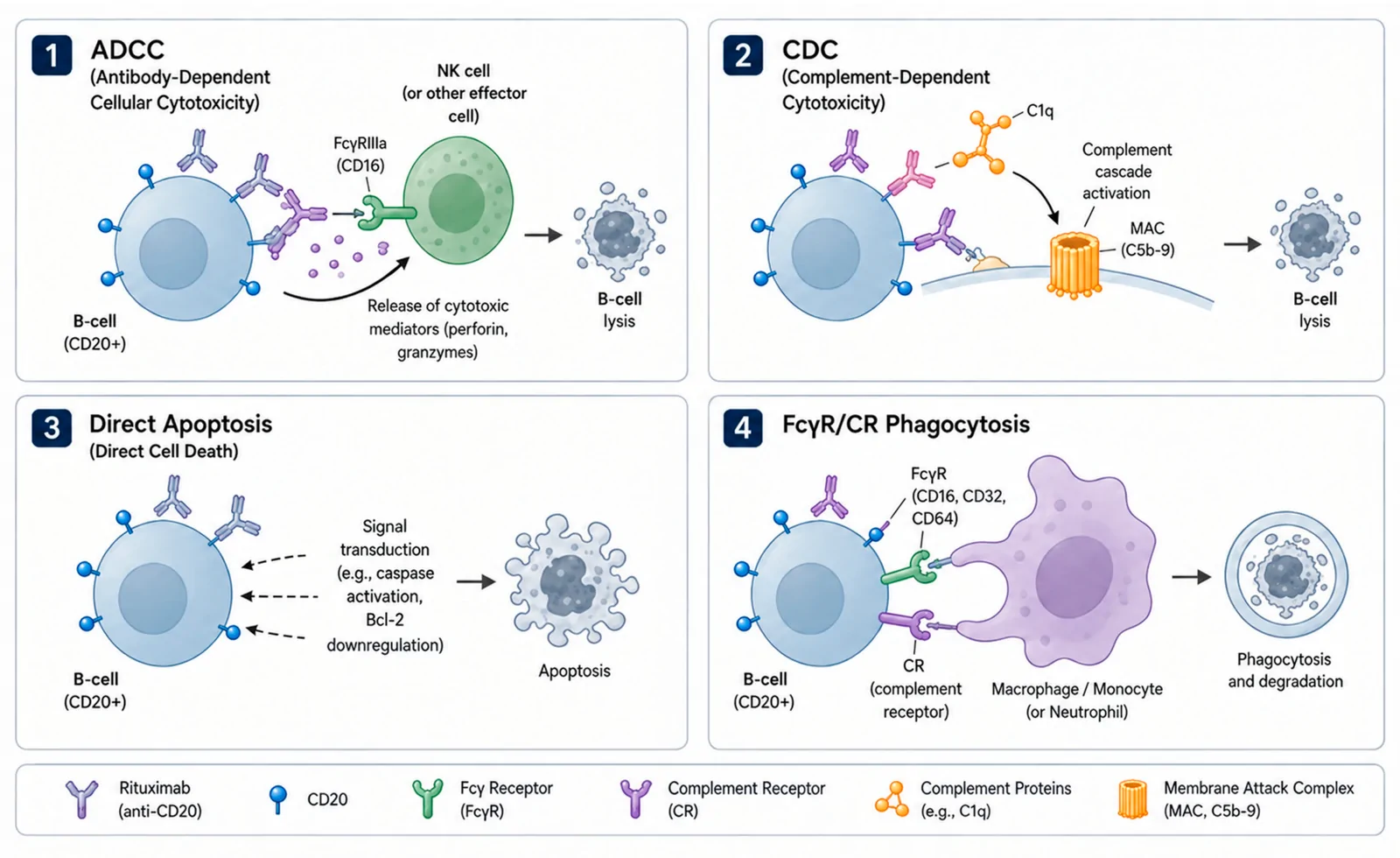
Rituximab’s mechanisms of action.

Given its high efficacy in the treatment of pemphigus vulgaris, rituximab has emerged as an attractive off-label therapeutic option for MMP. It binds to CD20 on the surface of late pro B cells and mature B cells, triggering antibody- and complement-mediated cytotoxicity, as well as direct apoptosis (Figure 2) [47]. Rituximab’s mechanism of action is far more complex than mere B-cell depletion. It suppresses the function of autoreactive T cells, promotes the activity of T-regulatory cells, and induces the production of B-cell activating factor, which supports long-lived, non-autoreactive plasma cells [47,48].

Although its mechanistic basis provides a strong rationale for its use in the treatment of mucous membrane pemphigoid, clinical outcomes varied considerably across studies. Rituximab has been largely used as an adjuvant treatment in MMP [49,50].

The largest studies were conducted by Bohelay et al. and Jeffrey et al., including 109 and 33 patients with severe, refractory MMP, respectively [50,51]. Complete remission in the absence of concomitant immunosuppressive treatment was achieved in 85% and 97% of patients, and was maintained for a mean time of 19–25 months in 59% and 68%, respectively, after one or two cycles of rituximab administered according to the pemphigus vulgaris protocol and repeated after 6 months when needed [51,52]. Immunomodulatory treatment, mainly with dapsone, was continued until sustained remission. In their 2022 systematic review, Lytvyn et al. identified 112 reported MMP cases treated with rituximab, which induced complete remission in 70.5% of patients, sustained throughout the variable follow-up period in 64.3% of cases [53]. Comparable outcomes were observed across multiple case series [54,55,56,57,58]. Nevertheless, different treatment protocols were used across studies and, in most reports, complete and partial remission were not clearly defined. In addition, the differential response of individual mucosal sites was not evaluated. The follow-up period varied widely, and relapses were not defined by the emergence of new blisters and ulcerations or by the progression of scarring. The dynamics of CD20+ B-cell number and autoantibody titers were seldom monitored. Consequently, the available evidence does not allow for a comprehensive assessment of the effects of rituximab on the clinical course of MMP. Several important issues require further clarification.

First and foremost, the optimal combination therapy—consisting of systemic corticosteroids and immunosuppressive or immunomodulatory agents—for each clinical form of MMP is yet to be determined. Based on the current literature, ocular and laryngeal MMP are associated with a poor response to both conventional and biologic treatment [46,59]. In their review of 167 reported cases of rituximab-treated MMP, Baniel et al. found that lesions located at different anatomic sites did not respond uniformly [60]. According to their analysis, complete response was achieved at a significantly lower frequency in laryngeal MMP than at other affected sites (40% vs. 67–80%) [60]. Additional cycles of rituximab proved ineffective in some recalcitrant laryngeal MMP cases; the majority of patients required chronic treatment with conventional immunosuppressive agents, IVIG, immunoadsorption, or a combination of these therapies [60]. A possible explanation is the late initiation of rituximab treatment, after irreversible scarring had occurred. It has also been demonstrated that in MMP, progression of scarring may continue even after the remission of inflammation [60,61]. From this perspective, early initiation of rituximab therapy, associated with IVIG, and close monitoring may represent a reasonable therapeutic strategy for patients with laryngeal MMP.

This observation brings into focus a second key issue that merits consideration, namely the most appropriate timing of rituximab therapy. Although the need for aggressive management in severe disease is evident, the role of rituximab in mild, apparently benign forms remains debated, with some authors advocating for its earlier use as even less severe forms of MMP often follow an unfavorable course, thus proving treatment resistance [52,60,61]. In 2022, Dastmalchi et al. reported their experience with rituximab in the treatment of MMP and compared the impact of early versus late administration on clinical outcomes. The investigators observed that patients receiving RTX earlier in the disease course achieved disease control sooner. As a result, the authors recommend rituximab as a first-line treatment to prevent function-impairing and life-threatening complications [58].

Thirdly, the most effective protocol for rituximab treatment in MMP remains undefined. Rituximab has been administered according to either the lymphoma protocol, i.e., 4 weekly administrations of 375 mg/m^2^, or the pemphigus vulgaris protocol, namely two doses of 1000 mg administered 2 weeks apart. Several authors have drawn contradictory conclusions [38,62]. Given the heterogeneity of the studies performed so far, it remains unclear which interpretation is correct. Notably, some experts advocate the use of higher doses of rituximab in severe, refractory MMP [55,63].

Given the recurrent nature of the disease, an effective maintenance treatment strategy is mandatory. Following rituximab treatment, B-cell repopulation of the peripheral blood occurs after a median period of 6 months. However, this is not always accompanied by disease relapse. Prolonged remissions are frequently observed, often extending beyond the period of B-cell depletion. This phenomenon may be explained by the immunomodulatory effects of rituximab on adaptive immune responses, including reductions in autoreactive T-cell populations and increases in regulatory B-cell numbers. Moreover, the B cells that repopulate the peripheral blood after rituximab therapy are predominantly naïve B cells (CD19^+^ and CD27^−^) with an immature phenotype (CD5^+^ and CD38high). Consequently, autoantibody titers may remain reduced even after B-cell reconstitution [64]. On the other hand, autoreactive B-cell clones may persist in lymphoid tissues and may drive the re-emergence of pathogenic germinal center responses following B-cell reconstitution [65]. Hence, it is not yet clear whether subsequent doses should be administered at regular, 6-month intervals or based on each patient’s clinical evolution. In the absence of biomarkers predictive of disease relapse, we favor the second approach. Excessive cumulative exposure to rituximab greatly increases the risk of adverse effects due to prolonged immunosuppression [47]. Therefore, in our view, additional doses of rituximab should only be offered when clinically justified.

The factors contributing to primary non-response to rituximab also warrant further investigation. An interesting finding reported by He et al. is that IgA-producing plasma cells are resistant to rituximab [66]. This observation was confirmed by Lamberts et al., who investigated the effect of rituximab in a series of IgA-dominant MMP and noted that while IgG-producing CD20+ B cells were depleted by rituximab, IgA-producing plasma cells were not affected [67]. In their study of patients with rheumatoid arthritis, Mei et al. showed that IgA-secreting plasma cells were persistently present in both the peripheral blood and the gastrointestinal mucosa during the period of effective rituximab-induced B-cell depletion [68]. These plasma cells displayed a mucosal phenotype, suggesting that mucosal-resident pre-B cells are resistant to rituximab [68]. Novel therapeutic options are needed to eliminate autoreactive tissue-resident memory B cells that escape rituximab-mediated depletion.

Last but not least, safety concerns should be addressed. Although rituximab does not pose a higher risk of serious infections than conventional immunosuppressants, the combination of the two therapies increases the rates of infection and mortality compared to conventional immunosuppressants [67,69]. Patients should also be monitored for other potential secondary reactions to rituximab, including late-onset myelosuppression with neutropenia or lymphopenia, interstitial lung disease, arrhythmias, acute coronary syndromes, serum sickness, anaphylaxis, among other less frequent adverse effects [70].

Several studies have explored therapeutic alternatives for refractory MMP, such as topical anti-tumor necrosis factor (TNF)-α in ocular MMP (NCT 06926478); oral Janus kinase inhibitors; low-dose interleukin (IL)-2 (EU CTN: 2023-510297-14-00); complement inhibitors; and the anti-IL-4Rα therapy dupilumab [43]. Next-generation fully human anti-CD20 agents, bispecific anti-CD19 and anti-CD32b monoclonal antibodies, and BAFF inhibitors could also be beneficial in recalcitrant MMP [43].

Advancing our understanding of the inflammatory pathways underlying MMP is critical for uncovering new therapeutic targets. These discoveries may stimulate further investment in clinical research and support the initiation of randomized controlled trials addressing the unmet needs of patients with MMP.

## 5. Conclusions

Although rarely fatal, MMP is a chronic, relapsing, disabling disease. Prompt diagnosis and early, aggressive treatment in the inflammatory phase are essential to prevent scarring that may lead to devastating complications. Based on our experience, we support the early use of rituximab in high-risk patients with MMP.

Although the combination of rituximab and IVIG has been proposed as a potentially effective therapeutic strategy in previous reports, the limited use of IVIG in our cohort precludes drawing conclusions regarding the efficacy or superiority of this combination. Further studies are needed to clarify the potential role of IVIG in combination with rituximab in MMP cases refractory to systemic glucocorticoids and conventional immunosuppressive agents. Long-term monitoring of these patients is mandatory to detect disease reactivation and initiate appropriate interventions before clinical progression.

Despite encouraging evidence, robust confirmation from prospective randomized controlled studies is still required. Nevertheless, the rarity of MMP and its marked clinical heterogeneity pose significant obstacles to trial design and implementation. The lack of a standardized rituximab regimen and validated strategies for monitoring treatment response represents a critical unmet need. In the absence of comprehensive, harmonized evidence, the optimal management of MMP remains a complex and demanding clinical challenge.

## Figures and Tables

**Table 1:** Foster’s staging system for ocular MMP [46].

Stage	Characteristics
Stage I	Subconjunctival scarring and fibrosis
Stage II	Fornix shortening
Stage III	Formation of symblepharon
Stage IV	Formation of ankyloblepharon and keratinization of the ocular surface

**Table 2:** Demographic and clinical characteristics of patients.

No.	Gender	Age	Comorbidities	Time from Disease Onset to Diagnosis (Months)	Anatomic Sites Involved	Disease Complications
1	M	79	benign prostatic adenoma, osteoporosis	2	skin, oral, and nasal involvement	none
2	F	69	type 2 diabetes mellitus, dyslipidemia	8	ocular, oropharyngeal, esophageal, and genital involvement	esophageal stenosis, loss of vision in the right eye
3	F	80	essential arterial hypertension, sinus bradycardia, premature extrasystolic arrhythmias, ischemic coronary artery disease, cervical esophageal diverticulum, anxiety disorder	60	skin, ocular, and oropharyngeal involvement	vision impairment, dysphagia
4	F	71	insulin-dependent type 2 diabetes mellitus, dyslipidemia, essential arterial hypertension, history of ischemic stroke	1	ocular and oropharyngeal involvement	vision loss in the left eye
5	F	68	essential arterial hypertension, chronic peripheral venous insufficiency, chronic hepatitis B infection	1	ocular involvement	severe vision impairment
6	F	71	type 2 diabetes mellitus, dyslipidemia, essential arterial hypertension	1	skin, ocular, and oropharyngeal involvement	vision loss in the right eye, dysphagia
7	F	39	no comorbidities	120	ocular involvement	vision impairment
8	F	58	dyslipidemia, osteopenia	72	ocular involvement	vision loss in the left eye

**Table 3:** Treatment outcomes.

No.	Conventional Treatment Before and After Rituximab	Time from Disease Onset to Rituximab Treatment (Months)	Disease Course Following Rituximab	Follow-Up Period (Months)
1	corticosteroids, dapsone	N/A	N/A	19
2	corticosteroids, dapsone (ceased due to severe anemia), methotrexate	11	partial remission of left eye inflammation, vision loss in the right eye, persistent dysphagia	9
3	corticosteroids, dapsone	72	remission of ocular inflammation, residual dysphagia	21
4	corticosteroids, dapsone	10	remission of right eye inflammation, left eye enucleation	19
5	corticosteroids, dapsone (ceased due to severe anemia), azathioprine	N/A	N/A	4
6	corticosteroids, azathioprine (ceased due to gastrointestinal adverse effects), dapsone	107	partial remission of ocular and oropharyngeal inflammation, followed by a severe relapse 12 months after the initial cycle of rituximab, which required an additional course of rituximab	60
7	corticosteroids, mycophenolate mofetil (ceased due to inefficiency), dapsone (ceased due to inefficiency), methotrexate	122	partial remission of ocular inflammation	7
8	corticosteroids, IVIG, dapsone	264	partial remission of right eye inflammation	60

## Data Availability

The data presented in this study are available upon request from the corresponding author due to privacy and ethical restrictions.

## Abbreviations

The following abbreviations are used in this manuscript:

MMP	Mucous membrane pemphigoid
